# Technological Advances in the Diagnosis and Management of Tinnitus

**DOI:** 10.3390/jcm11154597

**Published:** 2022-08-07

**Authors:** Jose Antonio Lopez-Escamez, Patricia Perez-Carpena

**Affiliations:** 1 Division of Otolaryngology, Department of Surgery, Facultad de Medicina, Parque Tecnológico de la Salud, Av. de la Investigación, 11, Universidad de Granada, 18016 Granada, Spain; 2 Otology & Neurotology Group CTS 495, Department of Genomic Medicine, GENYO, Centre for Genomics and Oncological Research, Pfizer/University of Granada/Andalusian Regional Government, PTS, 18016 Granada, Spain; 3 Sensorineural Pathology Programme, Centro de Investigación Biomédica en Red en Enfermedades Raras (CIBERER), 28029 Madrid, Spain; 4 Department of Otolaryngology, Instituto de Investigación Biosanitaria ibs.Granada, Hospital Universitario Virgen de las Nieves, Universidad de Granada, 18014 Granada, Spain

Tinnitus disorder is a bothersome perception of a composite noise or tone in the ears in the absence of an external source, associated with emotional distress, cognitive dysfunction, and/or autonomic arousal. Tinnitus frequently occurs as a symptom together with other hearing disorders (high-frequency hearing loss, Meniere’s disease, vestibular schwannoma), auditory pathway disorders (hyperacusis), mental disorders (anxiety, depression), vascular risk factors (high blood pressure, dyslipidemia), or neurological disorders (intracranial hypertension, migraine).

The condition may become a severe issue and lead to behavioural changes and functional disability, requiring a multidisciplinary intervention with different healthcare professionals including an audiologist, neurotologist, psychologist, and psychiatrist. The challenge is to improve the diagnostic procedures of tinnitus and assign the appropriate treatment or combination of treatments to each patient. 

A deep clinical characterization of patients with tinnitus is essential to identify which individuals have or show a high risk of developing tinnitus disorder. *Allgaier* et al. used *TrackYourTinnitus*, an mHealth platform, to generate prospective longitudinal data and investigate the tinnitus profile, including arousal, loudness, mood, and stress [1]. They found that tinnitus varies with temperature in certain countries and certain seasons. Interestingly, the highest probability of tinnitus occurred in the US, with an average chance of 87%, while the lowest probability was found in Switzerland, with 68%. They reported that tinnitus occurred the least in Switzerland in March (53%), and the most in the UK in August (98%).

Many epidemiological and genetic studies on tinnitus use self-reported data and some critical opinions have emerged regarding the reliability of self-reported data. *Haro-Hernandez* et al. compared online and hospital responses to the Spanish version of the European School for Interdisciplinary Tinnitus Research screening-questionnaire (ESIT-SQ) in tinnitus individuals using an unsupervised age clustering [2]. Hyperacusis was more frequent in the online survey, and it was perceived to be more severe in older individuals. The authors concluded that self-reported tinnitus surveys are a low-confidence source for tinnitus phenotyping, since no medical diagnosis can be confirmed, and additional clinical evaluation is needed for tinnitus research to reach diagnosis. Age-based cluster analysis might help to better define clinical profiles and compare responses between different subgroups of tinnitus patients.

The characterization of patients with tinnitus must include a complete audiological assessment. *Kang* et al. analyzed the clinical and audiological profile of patients with chronic tinnitus and noise-induced hearing loss or presbycusis [3]. They reported higher hearing thresholds, a smaller loudness of tinnitus, and a lower degree of damage to outer hair cells in patients with presbycusis than those with noise-induced hearing loss. Moreover, wave I and III latencies were more prolonged in patients with presbycusis, despite the lower hearing thresholds reported in this cohort. These findings may reflect the effects of aging in the auditory system and the audiological and clinical differences in young and old individuals with tinnitus [2].

The evidence to support effective treatment for tinnitus is very limited. The main reason for this is the limitations of randomized clinical trials (RCTs) on tinnitus, particularly the selection biases related to the patient heterogeneity. A systematic review of RCTs in patients with tinnitus have identified some of these pitfalls, and the authors provided a list of considerations to improve the design of RCTs in tinnitus patients [4]. They recommended assessing tinnitus duration and fluctuation, perceived annoyance, psychoacoustic profile, and hearing loss. A wide spectrum of therapeutic strategies, ranging from transcranial magnetic and vagus nerve stimulation to internet-based cognitive behavioural therapy or sound therapy, have been used. Some of the most common issues are the inability to blind patients with respect to control interventions, the small sample sizes, and the definition of unclear randomization methods. Taken together, RCTs in tinnitus field suffer from many methodological flaws, including a lack of strict and well-defined inclusion criteria, failure to eliminate or adjust by confounders such as hearing loss, stress, and tinnitus duration, variances in outcome measures and interventions, and barriers to the design.

A scoping review focused on the methodological aspects of the clinical studies evaluating hearing aids (HA) fitting as a part of tinnitus management [5]. Although all studies show that HA fitting has a positive impact on tinnitus perception in patients with hearing loss, the methodological heterogeneity (low sample size, lack of strict age inclusion criteria) does not allow for robust conclusions.

Two different studies conducted in Germany and South Korea also evaluated the psychological effect of HA fitting [6] and the satisfaction with amplification in daily life [7]. Hearing therapy lastingly improved tinnitus-related distress in mildly distressed German patients with chronic tinnitus and mild-to-moderate hearing loss. Korean individuals showed improvement in visual analog scale and Tinnitus Handicap Inventory scores and the authors concluded that patients with hearing loss and tinnitus can be treated with hearing aids and counseling. However, both were non-controlled studies; the authors measured the effect of the intervention before and after HA fitting, and the placebo effect could not be ruled out.

Educational counseling is a treatment approach that aims to educate tinnitus patients and inform them about possible coping strategies. *Schlee* et al. implemented educational material and self-help advice in a smartphone app [8]. Participants used the educational smartphone app unsupervised during their daily routine over a period of four months. The authors found that frequent and intensive use of the app was a crucial factor for treatment success, and participants that used the app more often and interacted with the app intensively reported a stronger improvement in their tinnitus.

Repetitive transcranial magnetic stimulation (rTMS) is a non-invasive, neuromodulating technique for brain hyperexcitability disorders and has been used in patients with chronic tinnitus. In a narrative review, *Denton* et al. [9] summarize the procedures used (low frequency during 10 consecutive sessions), the most targeted location (auditory cortex) and the overall efficacy of all the reviewed studies. Most of the RCTs have a low sample size and conflicting results. There was one systematic review and three meta-analyses of RCTs regarding the efficacy of rTMS for tinnitus treatment. Three out of the four reviews identified by the authors observed significant improvements in tinnitus with rTMS treatment compared to the outcomes observed in those receiving sham treatment. However, the heterogeneity among the current treatment protocols may limit the understanding of the effect of rTMS on tinnitus.

A more exciting approach, using electrical stimulation to suppress tinnitus, has been reported by *Poels* et al. [10]. Electrical stimulation with cochlear implants can significantly suppress the tinnitus sensations in 25–72% of implanted patients. However, no predictors for the effectiveness of tinnitus suppression with cochlear implants have been found, which substantially limits the potential application of cochlear implants for this purpose. The authors propose a trial using electrical round window stimulation (RWS) with local anesthesia as a diagnostic tool to identify candidates in whom electrical stimulation would be successful as a tinnitus treatment. They report complete tinnitus suppression in 77% of patients, and partial suppression in 23% of individuals, and conclude that this test could be used to identify tinnitus patients that would benefit from cochlear implantation.

The landscape of tinnitus diagnosis and management is changing very quickly, with technological advances and new opportunities for personalized medicine. The combination of machine learning and the use of smartphones to improve diagnosis, and the development of new electrical and magnetic stimulation techniques for personalized therapies, offer promising horizons for patients.

## References

[1] Allgaier J., Schlee W., Probst T., Pryss R. (2022). Prediction of Tinnitus Perception Based on Daily Life MHealth Data Using Country Origin and Season. J. Clin. Med..

[2] Haro-Hernandez E., Perez-Carpena P., Unnikrishnan V., Spiliopoulou M., Lopez-Escamez J.A. (2022). Standardized Clinical Profiling in Spanish Patients with Chronic Tinnitus. J. Clin. Med..

[3] Kang H.J., Kang D.W., Kim S.S., Oh T.I., Kim S.H., Yeo S.G. (2021). Analysis of Chronic Tinnitus in Noise-Induced Hearing Loss and Presbycusis. J. Clin. Med..

[4] Kikidis D., Vassou E., Schlee W., Iliadou E., Markatos N., Triantafyllou A., Langguth B. (2021). Methodological Aspects of Randomized Controlled Trials for Tinnitus: A Systematic Review and How a Decision Support System Could Overcome Barriers. J. Clin. Med..

[5] Kikidis D., Vassou E., Markatos N., Schlee W., Iliadou E. (2021). Hearing Aid Fitting in Tinnitus: A Scoping Review of Methodological Aspects and Effect on Tinnitus Distress and Perception. J. Clin. Med..

[6] Boecking B., Rausch L., Psatha S., Nyamaa A., Dettling-Papargyris J., Funk C., Brueggemann P., Rose M., Mazurek B. (2022). Hearing Therapy Improves Tinnitus-Related Distress in Mildly Distressed Patients with Chronic Tinnitus and Mild-to-Moderate Hearing Loss: A Randomized-Controlled Cross-Over Design. J. Clin. Med..

[7] Lee H.J., Kang D.W., Yeo S.G., Kim S.H. (2022). Hearing Aid Effects and Satisfaction in Patients with Tinnitus. J. Clin. Med..

[8] Schlee W., Neff P., Simoes J., Langguth B., Schoisswohl S., Steinberger H., Norman M., Spiliopoulou M., Schobel J., Hannemann R. (2022). Smartphone-Guided Educational Counseling and Self-Help for Chronic Tinnitus. J. Clin. Med..

[9] Denton A.J., Finberg A., Ashman P.E., Bencie N.B., Scaglione T., Kuzbyt B., Telischi F.F., Mittal R., Eshraghi A.A. (2021). Implications of Transcranial Magnetic Stimulation as a Treatment Modality for Tinnitus. J. Clin. Med..

[10] Poels L., Zarowski A., Leblans M., Vanspauwen R., van Dinther J., Offeciers E. (2021). Prognostic Value of Trial Round Window Stimulation for Selection of Candidates for Cochlear Implantation as Treatment for Tinnitus. J. Clin. Med..

